# 12621 Targeted Chemical-Genetic Screen Platform for Identifying Drug Modes-of-Action

**DOI:** 10.1017/cts.2021.661

**Published:** 2021-03-30

**Authors:** Kevin Lin, Maximilian Billmann, Henry Ward, Ya-Chu Chang, Anja-Katrin Bielinsky, Chad L. Myers

**Affiliations:** 1 Department of Computer Science and Engineering, University of Minnesota, Minneapolis, MN, USA; 2 Department of Biochemistry, Molecular Biology, and Biophysics, University of Minnesota, Minneapolis, MN, USA

## Abstract

ABSTRACT IMPACT: The key to advancing precision medicine is to deepen our understanding of drug modes-of-action (MOA). This project aims to develop a novel method for predicting MOA of potential drug compounds, providing an experimental and computational platform for more efficient drug discovery. OBJECTIVES/GOALS: To develop (1) a targeted CRISPR-Cas9 chemical-genetic screen approach, and (2) a computational method to predict drug mode-of-action from chemical-genetic interaction profiles. METHODS/STUDY POPULATION: Screening drugs against a gene deletion library can identify knockouts that modulate drug sensitivity. These chemical-genetic interaction (CGI) screens can be performed in human cell lines using a pooled lentiviral CRISPR-Cas9 approach to assess drug sensitivity/resistance of single-gene knockouts across the human genome. A targeted, rather than genome-wide, library can enable scaling these screens across many drugs.

CGI profiles can be derived from phenotypic screen readouts. These profiles are analogous to genetic interaction (GI) profiles, which represent sensitivity/resistance of gene knockouts to a second gene knockout rather than a drug. To computationally predict a drug’s genetic target, we leverage the property that a drug’s CGI profile will be similar to its target’s GI profile. RESULTS/ANTICIPATED RESULTS: Five proof-of-principle screens will be conducted with compounds that have existing genome-wide profiles and well-characterized MOA. I will generate CGI profiles for these five compounds and identify genes that are drug-sensitizers or drug-suppressors. I will then evaluate whether targeted library screens can recapitulate the CGIs found in genome-wide screens. Finally, I will develop a computational tool to integrate these CGI profiles with GI profiles (derived from another project) to predict gene-level and bioprocess-level drug targets. These predictions (from both targeted and genome-wide profiles) will be benchmarked against a drug-target and drug-bioprocess standard. DISCUSSION/SIGNIFICANCE OF FINDINGS: This work will develop a scalable, targeted chemical-genetic screen approach to discovering how putative therapeutics work. The targeted screen workflow provides a method for higher-throughput drug screening. The computational pipeline provides a powerful tool for exploring the MOA of uncharacterized drugs or repurposing FDA-approved drugs.

